# Supplementary material for: Efficacy and optimal dosage of various exercises for migraine: a multilevel network and dose-response meta-analysis
Source: PeerJ. 2025 Oct 20;13:e20254. doi: 10.7717/peerj.20254 (PMC12548639; doi:10.7717/peerj.20254)
Supplement: Supplemental Information 3 [file peerj-13-20254-s003.docx]

****Efficacy and Optimal Dosage of Various Exercises for Migraine: A Multilevel Network and Dose-Response Meta-analysis****

**Supplementary Files**

**Contents**

| **Supplementary 1: Search Strategy** |
| --- |
| **Supplementary 2: Characteristics of Included Studies** |
| **Supplementary 3: Risk of Bias in Individual Studies** |
| **Supplementary 4: The details of CINeMA** |
| **Supplementary 5: node-splitting analysis** |
| **Supplementary 6: Funnel plot** |
| **Supplementary 7: Acceptability** |
| **Supplementary 8: Subgroup analysis** |
| **Supplementary 9: Dose-response relationship** |
| **Supplementary 10: Forest plot of each data point informing each arm** |
| **Supplementary 11: Model fitting effect** |

**Supplementary 1: Search Strategy**

## 2.1 MEDLINE

**Table S1 Search Strategy**

| **Search** | **Query** |
| --- | --- |
| #1 | "Migraine"[Mesh] |
| #2 | ((((((((((((((((((((Disorder, Migraine) OR (Disorders, Migraine)) OR (Migraine Disorder)) OR (Headache, Migraine)) OR (Headaches, Migraine)) OR (Migraine Headaches)) OR (Migraine Headache)) OR (Acute Confusional Migraine)) OR (Migraine, Acute Confusional)) OR (Status Migrainosus)) OR (Abdominal Migraine)) OR (Migraine, Abdominal)) OR (Cervical Migraine Syndrome)) OR (Migraine Syndrome, Cervical)) OR (Hemicrania Migraine)) OR (Migraine, Hemicrania)) OR (Migraine Variant)) OR (Variant, Migraine)) OR (Sick Headache)) OR (Headache, Sick)) OR (Headache) |
| #3 | ("Migraine"[Mesh]) OR ((((((((((((((((((((Disorder, Migraine) OR (Disorders, Migraine)) OR (Migraine Disorder)) OR (Headache, Migraine)) OR (Headaches, Migraine)) OR (Migraine Headaches)) OR (Migraine Headache)) OR (Acute Confusional Migraine)) OR (Migraine, Acute Confusional)) OR (Status Migrainosus)) OR (Abdominal Migraine)) OR (Migraine, Abdominal)) OR (Cervical Migraine Syndrome)) OR (Migraine Syndrome, Cervical)) OR (Hemicrania Migraine)) OR (Migraine, Hemicrania)) OR (Migraine Variant)) OR (Variant, Migraine)) OR (Sick Headache)) OR (Headache, Sick)) OR (Headache) |
| #4 | "Exercise"[Mesh] |
| #5 | (((((((((((((((((((((((((((((((Exercises) OR (Exercise, Physical)) OR (Exercises, Physical)) OR (Physical Exercise)) OR (Physical Exercises)) OR (Exercise, Aerobic)) OR (Aerobic Exercise)) OR (Aerobic Exercises)) OR (Exercises, Aerobic)) OR (Exercise, Isometric)) OR (Exercises, Isometric)) OR (Isometric Exercises)) OR (Isometric Exercise)) OR (Acute Exercise)) OR (Acute Exercises)) OR (Exercise, Acute)) OR (Exercises, Acute)) OR (Exercise Training)) OR (Exercise Trainings)) OR (Training, Exercise)) OR (Trainings, Exercise)) OR (Physical Activity)) OR (Activities, Physical)) OR (Activity, Physical)) OR (Physical Activities)) OR (Yoga)) OR (Pilates)) OR (Aquatic exercise)) OR (Qi gong)) OR (Wu qin xi)) OR (Stretch)) OR (Strength) Sort by: Most Recent |
| #6 | ("Exercise"[Mesh]) OR ((((((((((((((((((((((((((((((((Exercises) OR (Exercise, Physical)) OR (Exercises, Physical)) OR (Physical Exercise)) OR (Physical Exercises)) OR (Exercise, Aerobic)) OR (Aerobic Exercise)) OR (Aerobic Exercises)) OR (Exercises, Aerobic)) OR (Exercise, Isometric)) OR (Exercises, Isometric)) OR (Isometric Exercises)) OR (Isometric Exercise)) OR (Acute Exercise)) OR (Acute Exercises)) OR (Exercise, Acute)) OR (Exercises, Acute)) OR (Exercise Training)) OR (Exercise Trainings)) OR (Training, Exercise)) OR (Trainings, Exercise)) OR (Physical Activity)) OR (Activities, Physical)) OR (Activity, Physical)) OR (Physical Activities)) OR (Yoga)) OR (Pilates)) OR (Aquatic exercise)) OR (Qi gong)) OR (Wu qin xi)) OR (Stretch)) OR (Strength)) |
| #7 | ((Randomized controlled trial[Title/Abstract]) OR (Randomized[Title/Abstract])) OR (Placebo[Title/Abstract]) |
| #9 | #3 AND #6 AND #7 |

**Supplementary 2: Characteristics of Included Studies**

**Table S2 Characteristics of Included Studies**

| Country | Funded | n | % Female | Mean Age (SD/Range) | Session_duration(min) | Supervise | Treatment | Duration (weeks) | Intensity prescribed (METs) | Exercise dose (METs/week) | Timepoints available | Outcomes measured |
| --- | --- | --- | --- | --- | --- | --- | --- | --- | --- | --- | --- | --- |
| Alipouri2023(1) | | | | | | | | | | | | |
| Iran | N | 12 | 1.00 | 25.33 (2.87) | 30 | Y | Aerobic | 8 | 3 | 270 | Post | VAS |
| Iran | N | 12 | 1.00 | 27.25 (4.63) | 0 | NA | Control | 0 | 0 | 0 | Post | VAS |
| Aslani2022(2) | | | | | | | | | | | | |
| Iran | Y | 12 | 1.00 | 30.25 (6.1) | 50 | Y | Resistance | 8 | 3 | 450 | Post | VAS |
| Iran | Y | 8 | 1.00 | 30.1 (7) | 0 | NA | Control | 0 | 0 | 0 | Post | VAS |
| Bond2018(3) | | | | | | | | | | | | |
| USA | Y | 54 | 1.00 | 38.5 (7.4) | 40 | Y | Aerobic | 16 | 4 | 480 | Post | HIT-6, NRS |
| USA | Y | 56 | 1.00 | 40 (8.4) | 0 | NA | Control | 0 | 0 | 0 | Post | HIT-6, NRS |
| Boroujeni2014(4) | | | | | | | | | | | | |
| Iran | Y | 21 | 1.00 | 35.4 (7.9) | 75 | Y | Yoga | 12 | 2 | 518 | Post | VAS |
| Iran | Y | 21 | 1.00 | 34.9 (8.37) | 0 | NA | Control | 0 | 0 | 0 | Post | VAS |
| Butt2022(5) | | | | | | | | | | | | |
| Pakistan | N | 14 | 0.85 | 33.2 (11.69) | 50 | Y | Aerobic | 12 | 4 | 400 | Post, 6 weeks | HIT-6, NRS |
| Pakistan | N | 14 | 0.85 | 26.21 (6.89) | 0 | NA | Control | 0 | 0 | 0 | Post, 6 weeks | HIT-6, NRS |
| Darabanea2011(6) | | | | | | | | | | | | |
| USA | Y | 8 | NA | NA (NA) | 30 | Y | Aerobic | 10 | 4 | 360 | Post | NRS |
| USA | Y | 8 | NA | NA (NA) | 0 | NA | Control | 0 | 0 | 0 | Post | NRS |
| Eslami2021(7) | | | | | | | | | | | | |
| Iran | Y | 15 | 1.00 | 38.41 (6.2) | 40 | Y | Aerobic | 8 | 4 | 360 | Post | VAS |
| Iran | Y | 15 | 1.00 | 25.16 (6.08) | 40 | Y | HIIT | 8 | 7 | 840 | Post | VAS |
| Iran | Y | 15 | 1.00 | 32.44 (5.74) | 0 | NA | Control | 0 | 0 | 0 | Post | VAS |
| John2007(8) | | | | | | | | | | | | |
| India | Y | 36 | 0.45 | 34.38 (8.74) | 60 | Y | Yoga | 12 | 2 | 690 | Post | T-PRI, VAS |
| India | Y | 36 | 0.18 | NA (NA) | 0 | NA | Control | 0 | 0 | 0 | Post | T-PRI, VAS |
| Johnson2025(9) | | | | | | | | | | | | |
| USA | Y | 11 | 0.98 | 28.6 (4) | 30 | Y | Aerobic | 4 | 3 | 270 | Post | Migraine intensity scale |
| USA | Y | 9 | 0.96 | 32.9 (4) | 0 | NA | Control | 0 | 0 | 0 | Post | Migraine intensity scale |
| Kaushal2023(10) | | | | | | | | | | | | |
| India | N | 40 | 0.87 | 37.1 (10.2) | 30 | Y | Yoga | 3 | 2 | 345 | Post | HIT-6, VAS |
| India | N | 40 | 0.95 | 35.98 (8.69) | 0 | NA | Control | 0 | 0 | 0 | Post | HIT-6, VAS |
| Kisan2014(11) | | | | | | | | | | | | |
| India | Y | 47 | 0.70 | 31.72 (10.77) | 30 | Y | Yoga | 6 | 2 | 138 | Post | VAS |
| India | Y | 37 | 0.63 | 31.27 (8.63) | 0 | NA | Control | 0 | 0 | 0 | Post | VAS |
| Krøll2017(12) | | | | | | | | | | | | |
| Denmark | Y | 36 | 0.88 | 42 (10.9) | 45 | Y | Aerobic | 4 | 4 | 540 | Post, 12 weeks | NRS |
| Denmark | Y | 34 | 0.88 | 36 (10.1) | 0 | NA | Control | 0 | 0 | 0 | Post, 12 weeks | NRS |
| Kumar2020(13) | | | | | | | | | | | | |
| India | Y | 80 | 0.72 | 30.5 (8.01) | 30 | Y | Yoga | 8 | 2 | 345 | Post | HIT-6, NRS |
| India | N | 80 | 0.72 | 30.5 (8.01) | 30 | Y | Yoga | 8 | 2 | 207 | Post | HIT-6, NRS |
| India | Y | 80 | 0.66 | 31.9 (8.17) | 0 | NA | Control | 0 | 0 | 0 | Post | HIT-6, NRS |
| India | N | 80 | 0.66 | 31.9 (8.17) | 0 | NA | Control | 0 | 0 | 0 | Post | HIT-6, NRS |
| Kumari2022(14) | | | | | | | | | | | | |
| India | N | 23 | 1.00 | 33.17 (5) | 30 | Y | Yoga | 3 | 2 | 345 | Post | HIT-6, VAS |
| India | N | 20 | 1.00 | 33.2 (5) | 0 | NA | Control | 0 | 0 | 0 | Post | HIT-6, VAS |
| Lemstra2002(15) | | | | | | | | | | | | |
| Canada | Y | 44 | 0.72 | 35.59 (10.15) | 30 | Y | Aerobic | 6 | 4 | 480 | Post | VAS |
| Canada | Y | 44 | 0.72 | 35.59 (10.15) | 30 | Y | Aerobic | 6 | 4 | 360 | 12 weeks | VAS |
| Canada | Y | 36 | 0.58 | 33.17 (13.21) | 0 | NA | Control | 0 | 0 | 0 | Post, 12 weeks | VAS |
| Matin2022(16) | | | | | | | | | | | | |
| Iran | N | 16 | 1.00 | 30 (4) | 40 | Y | HIIT | 8 | 5 | 840 | Post | MIDAS |
| Iran | N | 16 | 1.00 | 30.5 (4) | 0 | NA | Control | 0 | 0 | 0 | Post | MIDAS |
| Mehta2021(17) | | | | | | | | | | | | |
| India | Y | 21 | 0.85 | 39.15 (8.24) | 30 | Y | stretch | 12 | 2 | 420 | Post | HIT-6, VAS |
| India | Y | 20 | 0.65 | 34.3 (9.57) | 30 | Y | Yoga | 12 | 2 | 483 | Post | HIT-6, VAS |
| India | Y | 20 | 0.65 | 34.3 (9.57) | 30 | Y | Yoga | 8 | 2 | 483 | Post | HIT-6, VAS |
| India | Y | 20 | 0.65 | 34.3 (9.57) | 30 | Y | Yoga | 4 | 2 | 483 | Post | HIT-6, VAS |
| India | Y | 20 | 0.71 | 36.81 (10.85) | 0 | NA | Control | 0 | 0 | 420 | Post | HIT-6, VAS |
| India | Y | 20 | 0.71 | 36.81 (10.85) | 0 | NA | Control | 0 | 0 | 0 | Post | VAS |
| Narin2003(18) | | | | | | | | | | | | |
| Turkey | N | 20 | 1.00 | 35 (5) | 50 | Y | Aerobic | 8 | 4 | 1000 | Post | QLS |
| Turkey | N | 20 | 1.00 | 40.1 (4) | 50 | Y | Aerobic | 8 | 3 | 450 | Post | VAS |
| Turkey | N | 20 | 1.00 | 50.1 (5) | 0 | NA | Control | 0 | 0 | 0 | Post | QLS, VAS |
| Turkey | N | 20 | 1.00 | 40 (5.5) | 0 | NA | Control | 0 | 0 | 0 | Post | QLS, VAS |
| Niu2024(19) | | | | | | | | | | | | |
| Turkey | N | 50 | 0.51 | 68.35 (5.29) | 35 | Y | Aerobic+Resistance | 24 | 4 | 420 | Post | HIT-6, MIDAS |
| Turkey | N | 50 | 0.51 | 66.42 (4.51) | 0 | NA | Control | 0 | 0 | 0 | Post | HIT-6, MIDAS |
| Oliveira2019(20) | | | | | | | | | | | | |
| Brazil | N | 15 | 0.84 | 36.2 (10.9) | 40 | Y | Aerobic | 12 | 3 | 720 | Post | Migraine intensity scale |
| Brazil | N | 15 | 0.76 | 36.2 (10.9) | 0 | NA | Control | 0 | 0 | 0 | Post | Migraine intensity scale |
| Ozge2025(21) | | | | | | | | | | | | |
| Turkey | N | 20 | 1.00 | 31.1 (4.5) | 30 | Y | Aerobic | 8 | 3 | 180 | Post | VAS |
| Turkey | N | 20 | 1.00 | 30.7 (3.8) | 20 | Y | stretch | 8 | 2 | 80 | Post | VAS |
| Turkey | N | 20 | 1.00 | 30.4 (4.21) | 0 | NA | Control | 0 | 0 | 0 | Post | VAS |
| Ozlem2024(22) | | | | | | | | | | | | |
| Turkey | N | 20 | 1.00 | 35.8 (7.78) | 45 | Y | Yoga | 12 | 2 | 310 | Post | HIT-6, MIDAS, VAS |
| Turkey | N | 20 | 1.00 | 36.65 (9.62) | 0 | NA | Control | 0 | 0 | 0 | Post | HIT-6, MIDAS, VAS |
| Peres2018(23) | | | | | | | | | | | | |
| Brazil | Y | 25 | 0.80 | 41.8 (19.7) | 30 | Y | Aerobic | 24 | 4 | 360 | Post | NRS |
| Brazil | Y | 25 | 0.84 | 41.1 (16.4) | 0 | NA | Control | 0 | 0 | 0 | Post | NRS |
| Rahimi2022(24) | | | | | | | | | | | | |
| Iran | N | 24 | 1.00 | 31.65 (6.4) | 30 | Y | Aerobic | 12 | 3 | 360 | Post, 54 weeks | VAS |
| Iran | N | 24 | 1.00 | 31.61 (4.87) | 0 | NA | Control | 0 | 0 | 0 | Post, 54 weeks | VAS |
| Sun2021(25) | | | | | | | | | | | | |
| China | Y | 145 | 0.81 | 33.1 (5.9) | 60 | Y | Resistance | 12 | 3 | 360 | Post, 16 weeks | VAS |
| China | Y | 141 | 0.83 | 34.5 (6.3) | 0 | NA | Control | 0 | 0 | 0 | Post, 16 weeks | VAS |
| Varkey2011(26) | | | | | | | | | | | | |
| Sweden | Y | 30 | 0.93 | 47 (10.8) | 30 | Y | Aerobic | 12 | 4 | 360 | Post, 12 weeks | VAS |
| Sweden | Y | 30 | 0.93 | 47 (10.8) | 40 | Y | Aerobic | 12 | 4 | 360 | 24 weeks | VAS |
| Sweden | Y | 30 | 0.83 | 41.5 (11.4) | 0 | NA | Control | 0 | 0 | 0 | Post, 12 weeks, 24 weeks | VAS |
| Xie2022(27) | | | | | | | | | | | | |
| China | Y | 42 | 1.00 | 50.9 (10.2) | 20 | Y | Tai Chi | 12 | 3 | 132 | Post, 24 weeks | VAS |
| China | Y | 40 | 1.00 | 47.1 (11.8) | 0 | NA | Control | 0 | 0 | 0 | Post, 24 weeks | VAS |

**Supplementary 3: Risk of Bias in Individual Studies**

**Table S3 Risk of Bias in Individual Studies**

| Author, Year | Bias arising from the randomization process | Bias due to deviations from intended interventions | Bias due to missing outcome data | Bias in measurement of the outcome | Bias in selection of the reported result | Overall bias |
| --- | --- | --- | --- | --- | --- | --- |
| Alipouri2023 | Low risk | Low risk | Low risk | Low risk | Low risk | Low |
| Aslani2022 | Some concerns | Some concerns | Some concerns | Some concerns | Some concerns | Unclear |
| Bond2018 | Low risk | Some concerns | Low risk | Low risk | Low risk | Unclear |
| Boroujeni2014 | Low risk | Some concerns | Some concerns | Some concerns | Low risk | Unclear |
| Butt2022 | Some concerns | High risk | Some concerns | Some concerns | Some concerns | High |
| Darabanea2011 | Low risk | Some concerns | Low risk | Low risk | Low risk | Unclear |
| Eslami2021 | Low risk | High risk | Some concerns | Some concerns | Some concerns | High |
| John2007 | Low risk | Some concerns | High risk | Some concerns | Some concerns | High |
| Johnson2025 | Low risk | Some concerns | Low risk | Some concerns | Low risk | Unclear |
| Kaushal2023 | Low risk | Some concerns | High risk | High risk | Some concerns | High |
| Kisan2014 | Low risk | Low risk | High risk | Some concerns | Some concerns | High |
| Krøll2017 | Low risk | Some concerns | Some concerns | Some concerns | Low risk | Unclear |
| Kumar2020 | Low risk | Low risk | Low risk | Low risk | Low risk | Low |
| Kumari2022 | Low risk | Some concerns | Low risk | Low risk | Low risk | Unclear |
| Lemstra2002 | Low risk | Low risk | Low risk | Low risk | Low risk | Low |
| Matin2022 | Low risk | Some concerns | Low risk | Low risk | Low risk | Unclear |
| Mehta2021 | Low risk | Low risk | Some concerns | Some concerns | Some concerns | Unclear |
| Narin2003 | Some concerns | Some concerns | Some concerns | High risk | Low risk | High |
| Niu2024 | Low risk | Some concerns | Low risk | Some concerns | Low risk | Unclear |
| Oliveira2019 | Low risk | Some concerns | High risk | High risk | High risk | High |
| Ozge2025 | Low risk | Some concerns | Low risk | Some concerns | Low risk | Unclear |
| Ozlem2024 | Low risk | Some concerns | Low risk | Some concerns | Low risk | Unclear |
| Peres2018 | Low risk | Some concerns | Some concerns | Some concerns | Some concerns | Unclear |
| Rahimi2022 | Low risk | Some concerns | Low risk | Some concerns | Low risk | Unclear |
| Sun2021 | Low risk | Some concerns | Low risk | Low risk | Low risk | Unclear |
| Varkey2011 | Low risk | Some concerns | Low risk | Some concerns | Low risk | Unclear |
| Xie2022 | Low risk | Low risk | Low risk | Low risk | Low risk | Low |


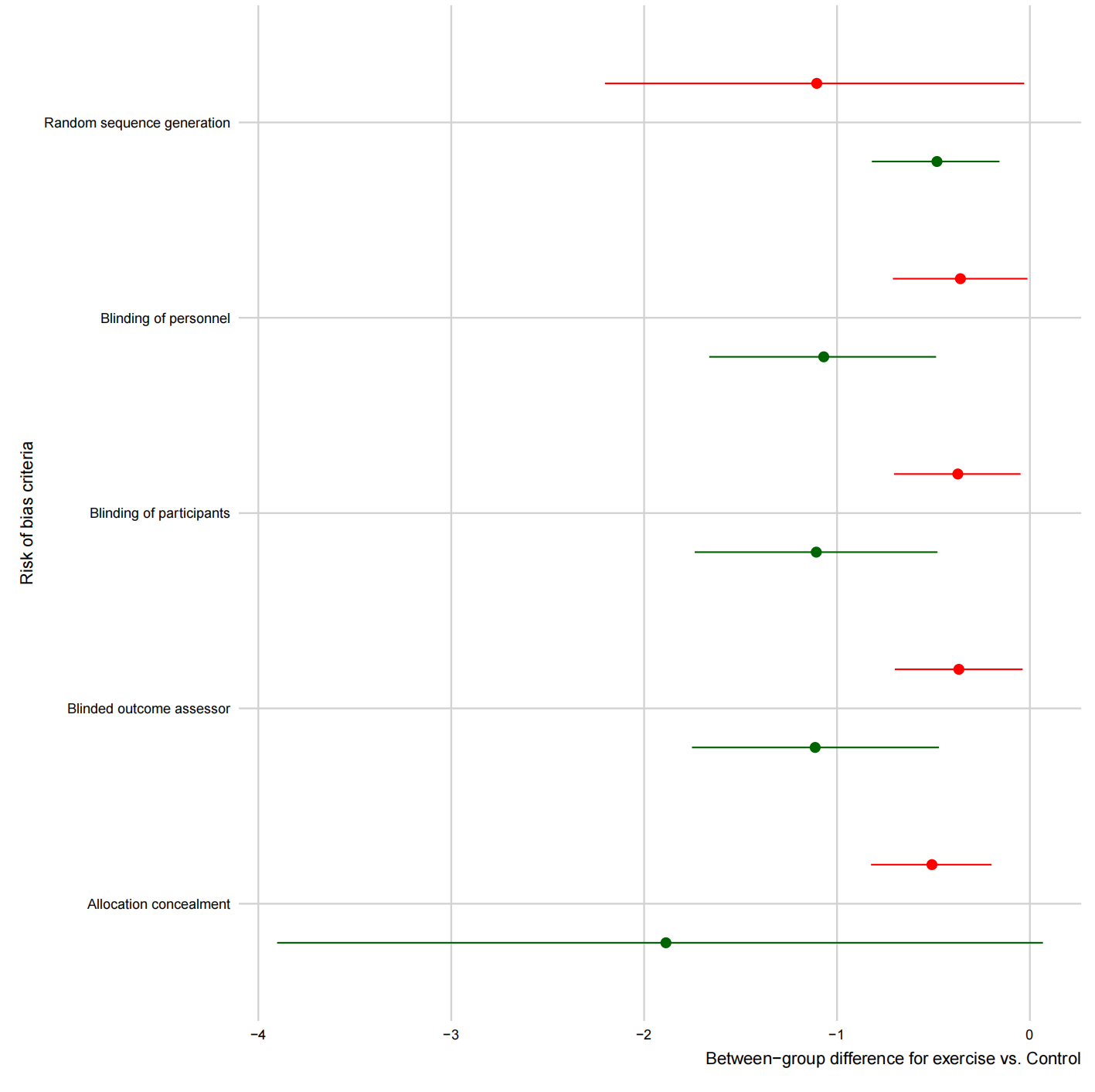


**Figure S1 Risk of Bias in Individual Studies**

**Supplementary 4: The details of CINeMA**

**Table S4. Summary table for credibility assessment using confidence in network meta-analysis (CINeMA)**

| **Control group versus** | **Direct**  **comparisons (k)** | **Within**  **study bias** | **Reporting**  **bias** | **Indirectness** | **Imprecision** | **Heterogeneity** | **Incoherence** | **Confidence**  **rating** |
| --- | --- | --- | --- | --- | --- | --- | --- | --- |
| Aerobic exercise | 16 | ● | ● | ● | ● | ● | ● | Low |
| Yoga | 9 | ● | ● | ● | ● | ● | ● | Low |
| Tai Chi | 1 | ● | ● | ● | ● | ● | ● | Very low |
| Resistance exercise | 2 | ● | ● | ● | ● | ● | ● | Low |
| Aerobic+resistance exercise | 1 | ● | ● | ● | ● | ● | ● | Low |
| HIIT | 2 | ● | ● | ● | ● | ● | ● | Very low |
| stretch exercise | 2 | ● | ● | ● | ● | ● | ● | Low |

**Risk of bias**

● Major concerns or high ● Some concerns or unclear ● No concerns or low

**Supplementary 5: node-splitting analysis**

**Table S5 node-splitting analysis**

| comparison | k | prop | NMA | Direct | indirect | Diff | z | p |
| --- | --- | --- | --- | --- | --- | --- | --- | --- |
| Aerobic : Control | 14 | 0.98 | –0.4017 | –0.3787 | –1.6750 | 1.2963 | 0.85 | 0.395 |
| Aerobic : HIIT | 1 | 0.53 | 0.2203 | –0.8965 | 1.464 | –2.3605 | –1.9 | 0.051 |
| Aerobic : stretch | 1 | 0.36 | –0.2928 | 0.221 | –0.5836 | 0.8046 | 0.79 | 0.430 |
| HIIT : Control | 2 | 0.86 | –0.6219 | –0.9625 | 1.5143 | –2.4769 | –1.48 | 0.139 |
| stretch : Control | 2 | 0.76 | –0.1089 | 0.2753 | –1.3411 | 1.6164 | 1.47 | 0.141 |
| Yoga : Control | 8 | 0.98 | –0.4905 | –0.4857 | –0.7597 | 0.274 | 0.14 | 0.885 |
| stretch : Yoga | 1 | 0.51 | 0.3817 | 0.1352 | 0.6395 | –0.5042 | –0.51 | 0.609 |

**Supplementary 6: Funnel plot**


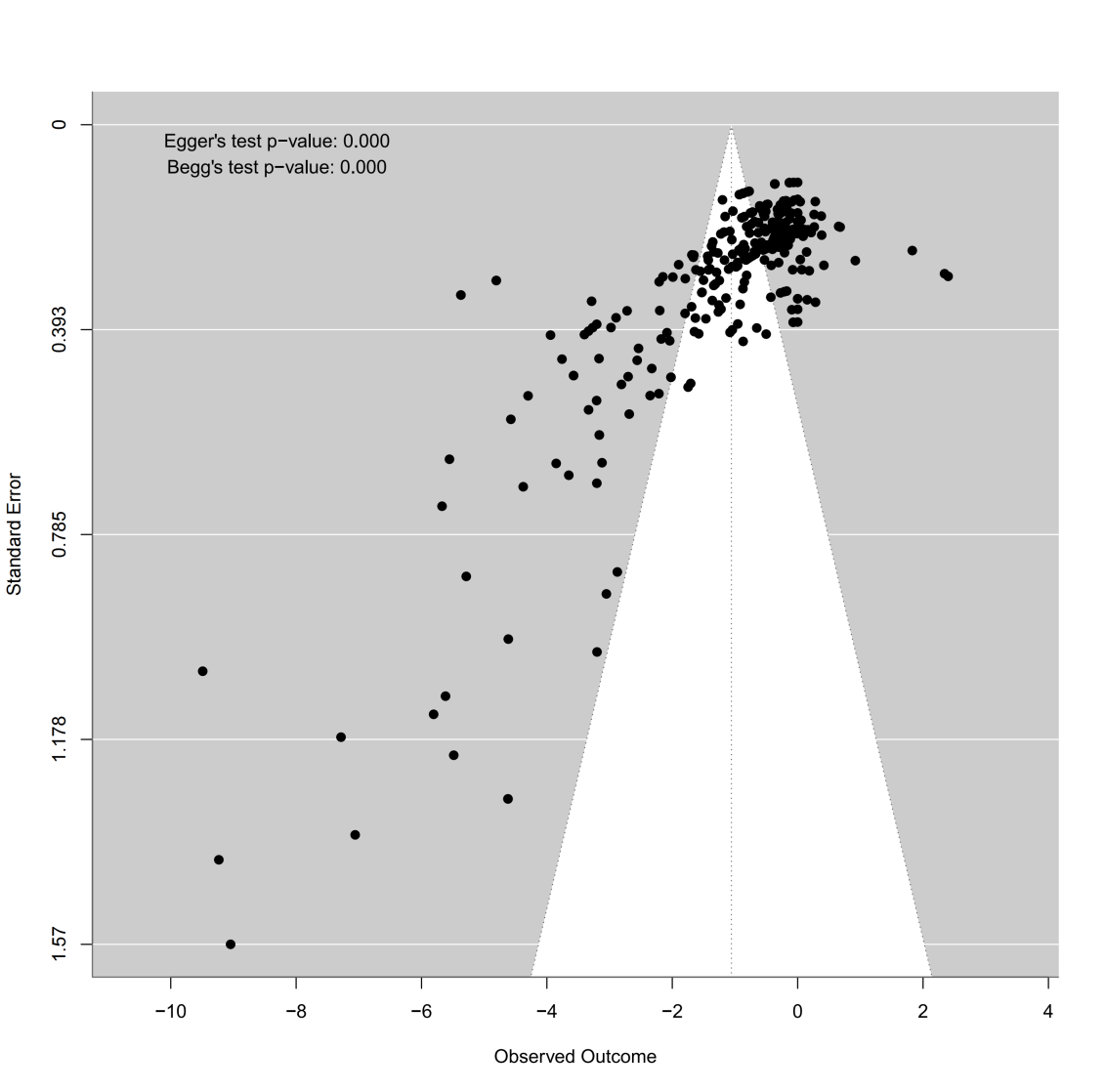


**Figure S2 Funnel plots**

**Supplementary 7: Acceptability**


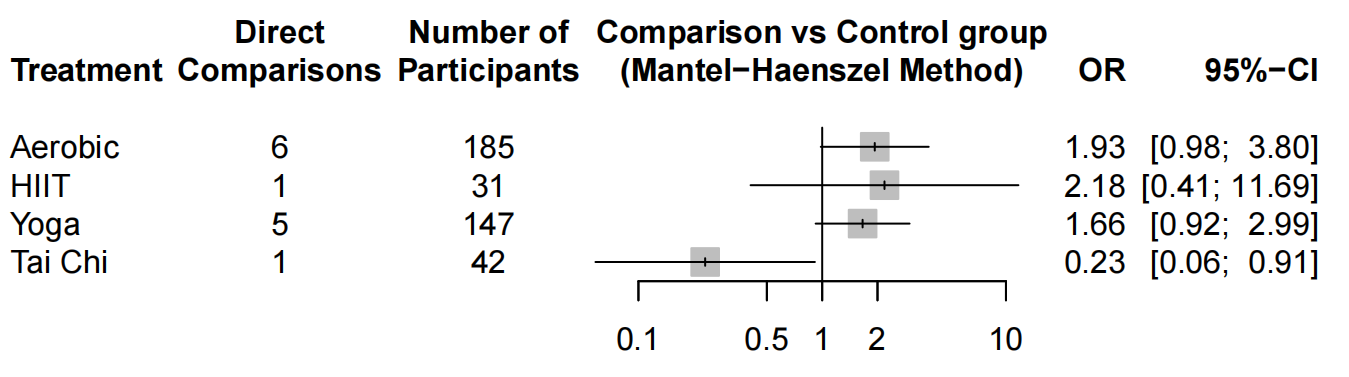


**Figure S3 Acceptability**

**Supplementary 8: Subgroup analysis**


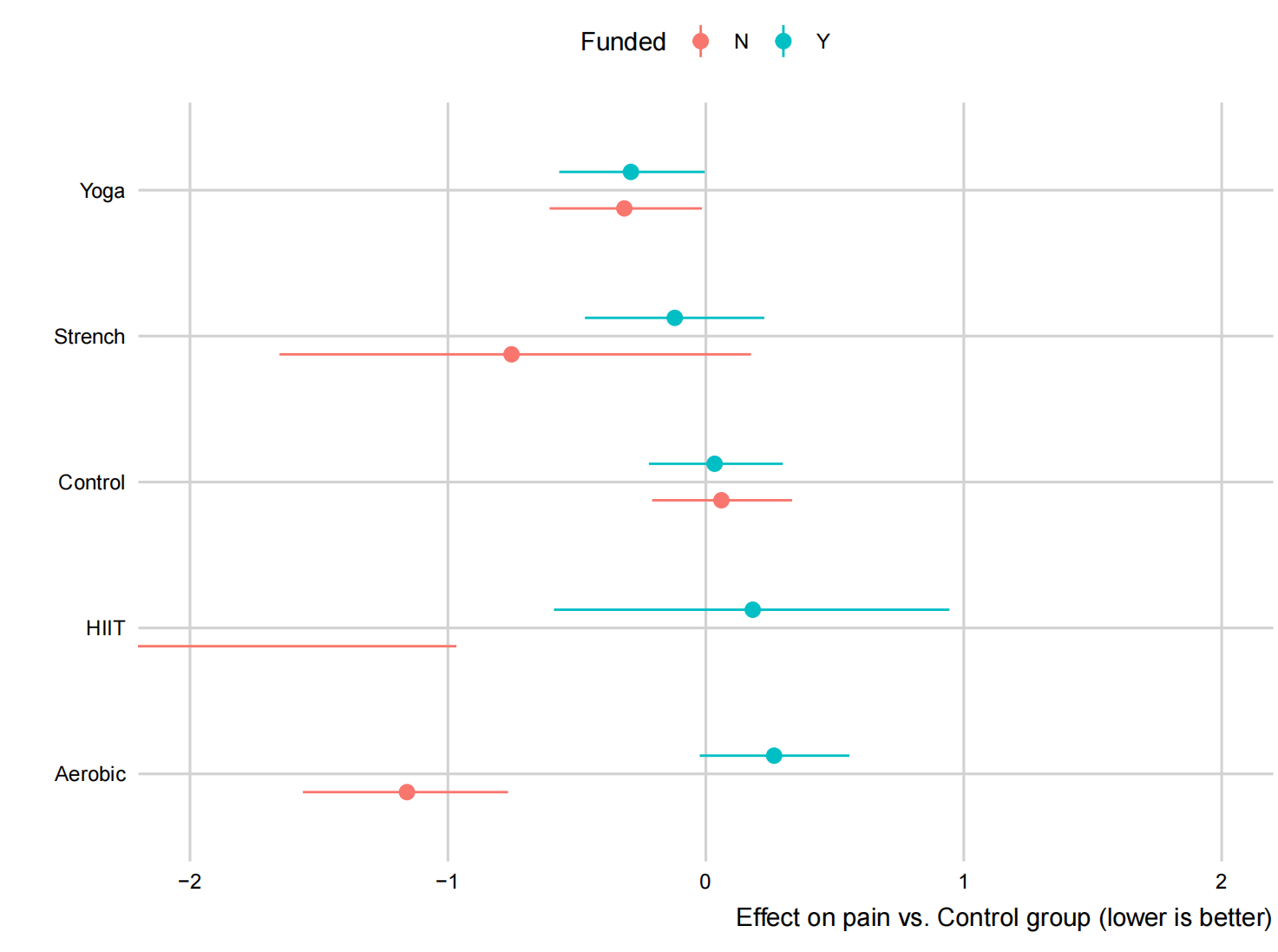


**Figure S4(a) Moderation by funded**


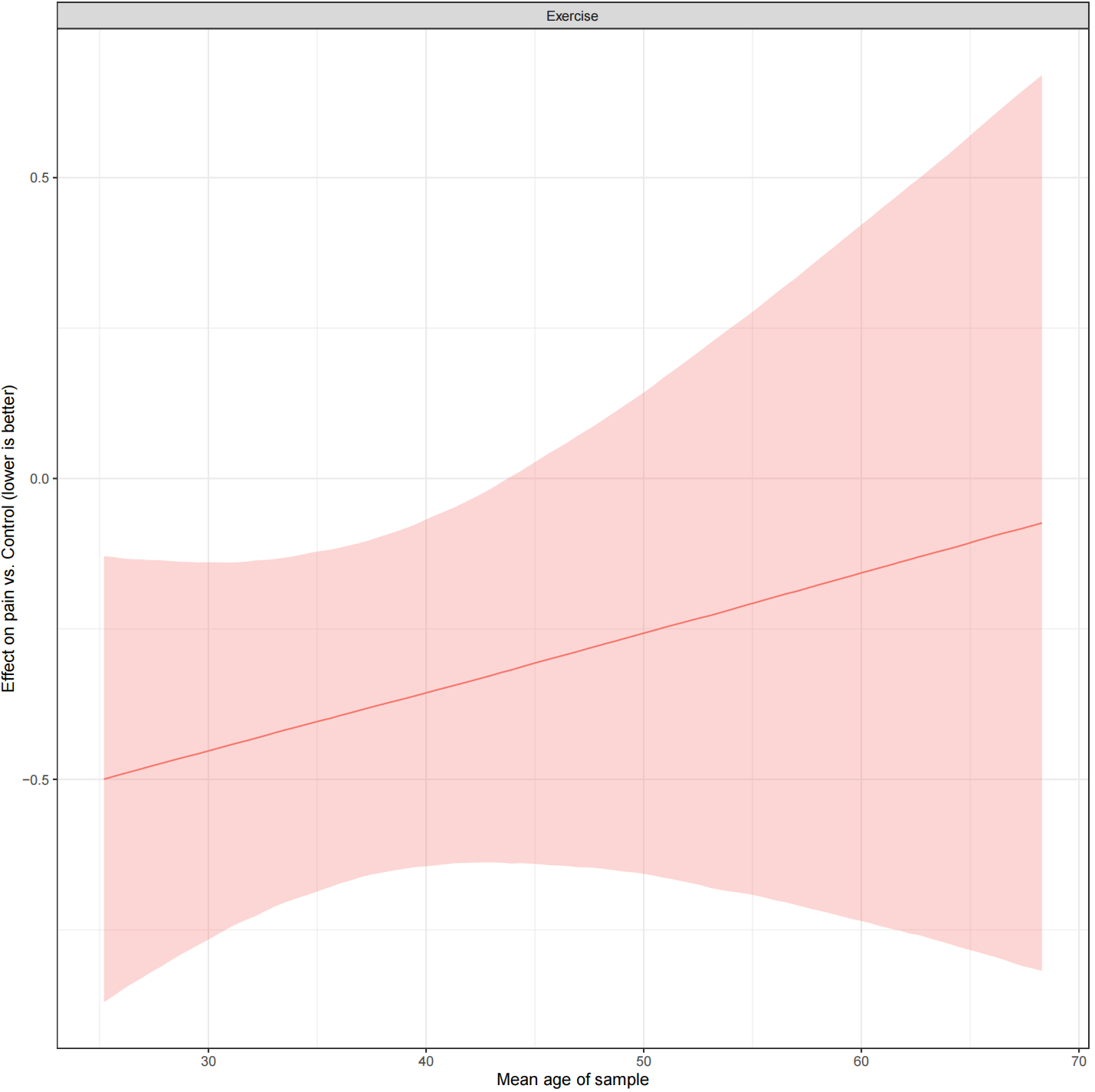


**Figure S4(b) Moderation by age**


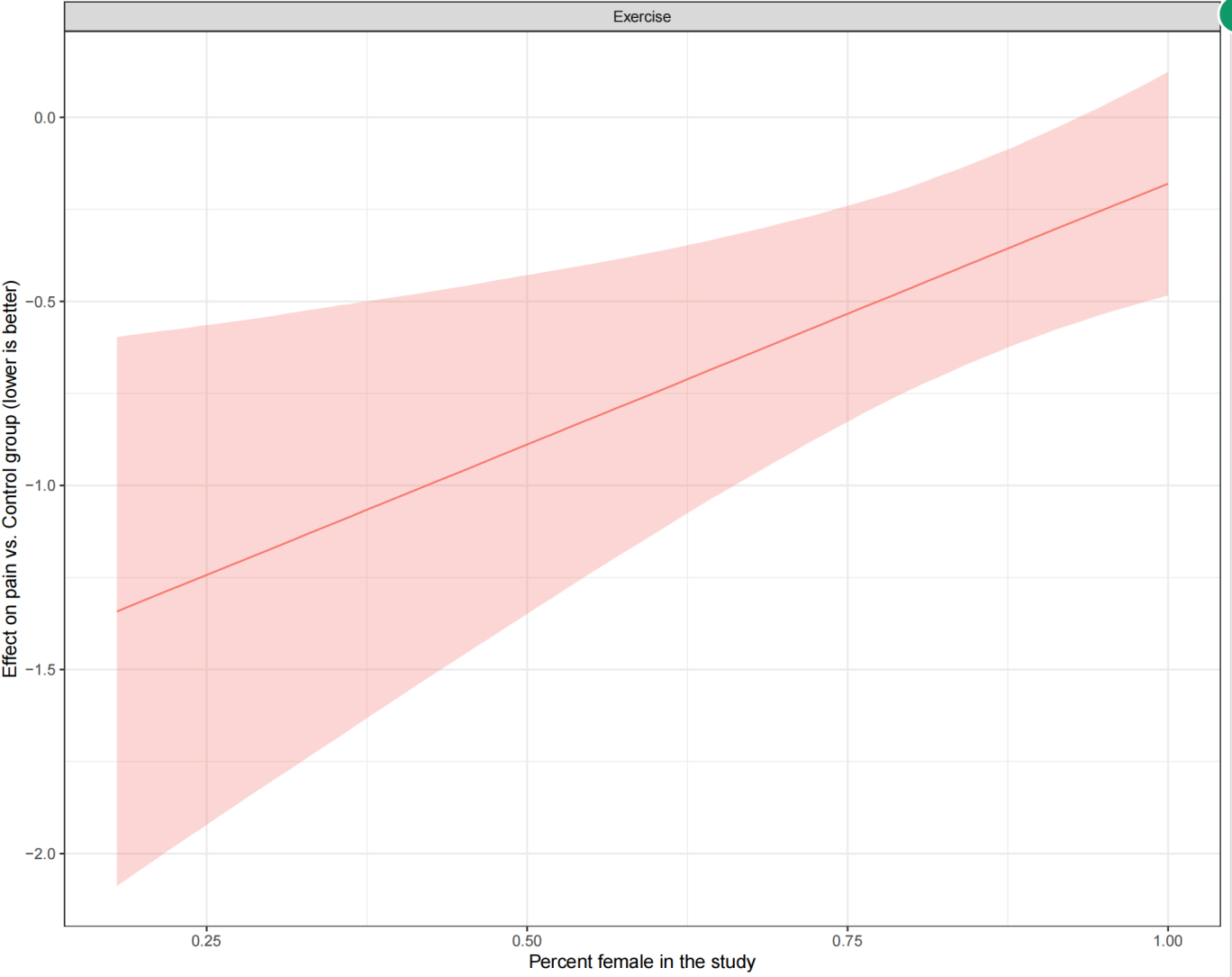


**Figure S4(c) Moderation by sex**

**Supplementary 9: Dose-response relationship**


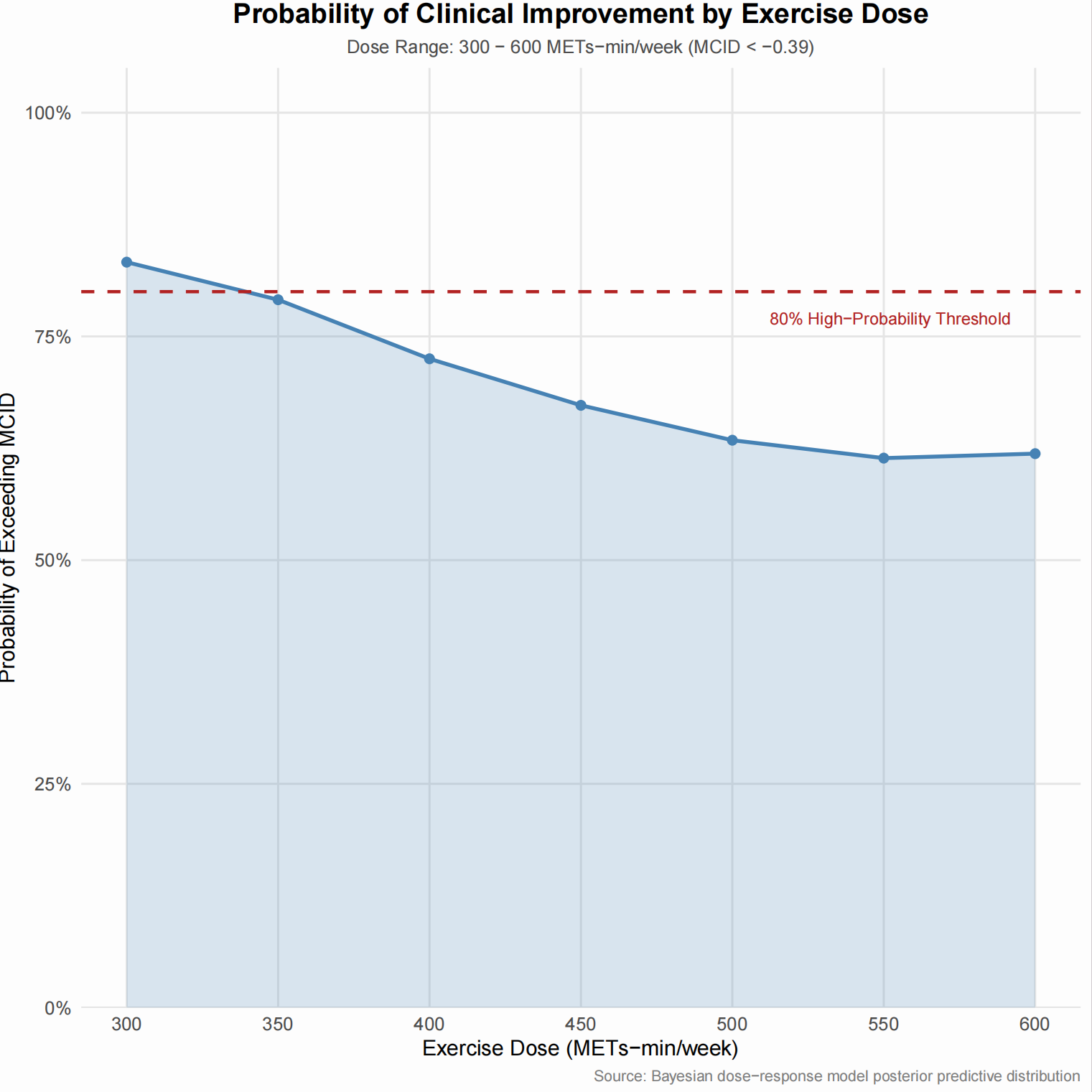


**Figure S5(a) Probability of Clinical Improvement by Exercise Dose**


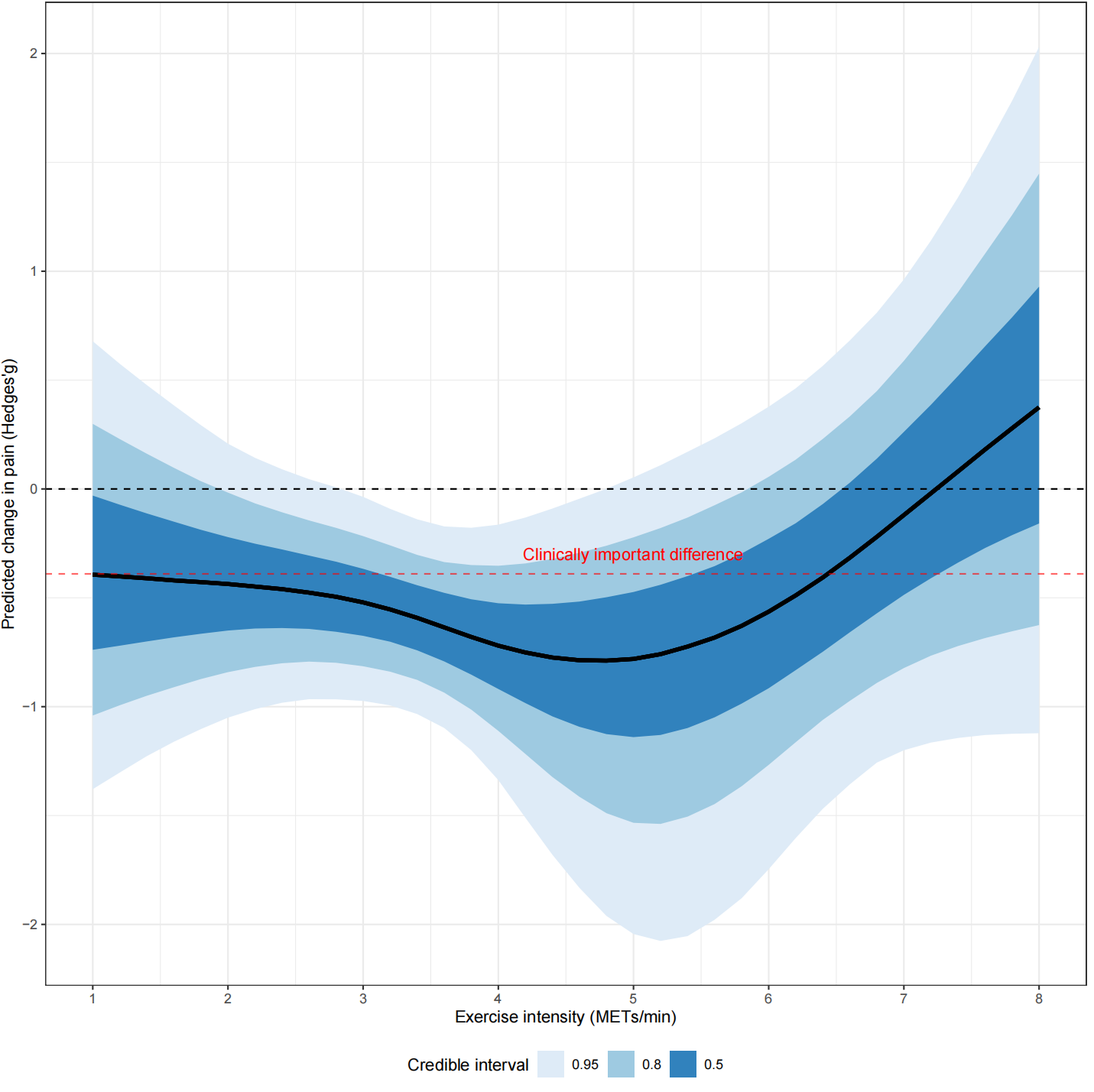


**Figure 5(b) Dose-response relationship of exercise intensity (Minutes) to migraine**


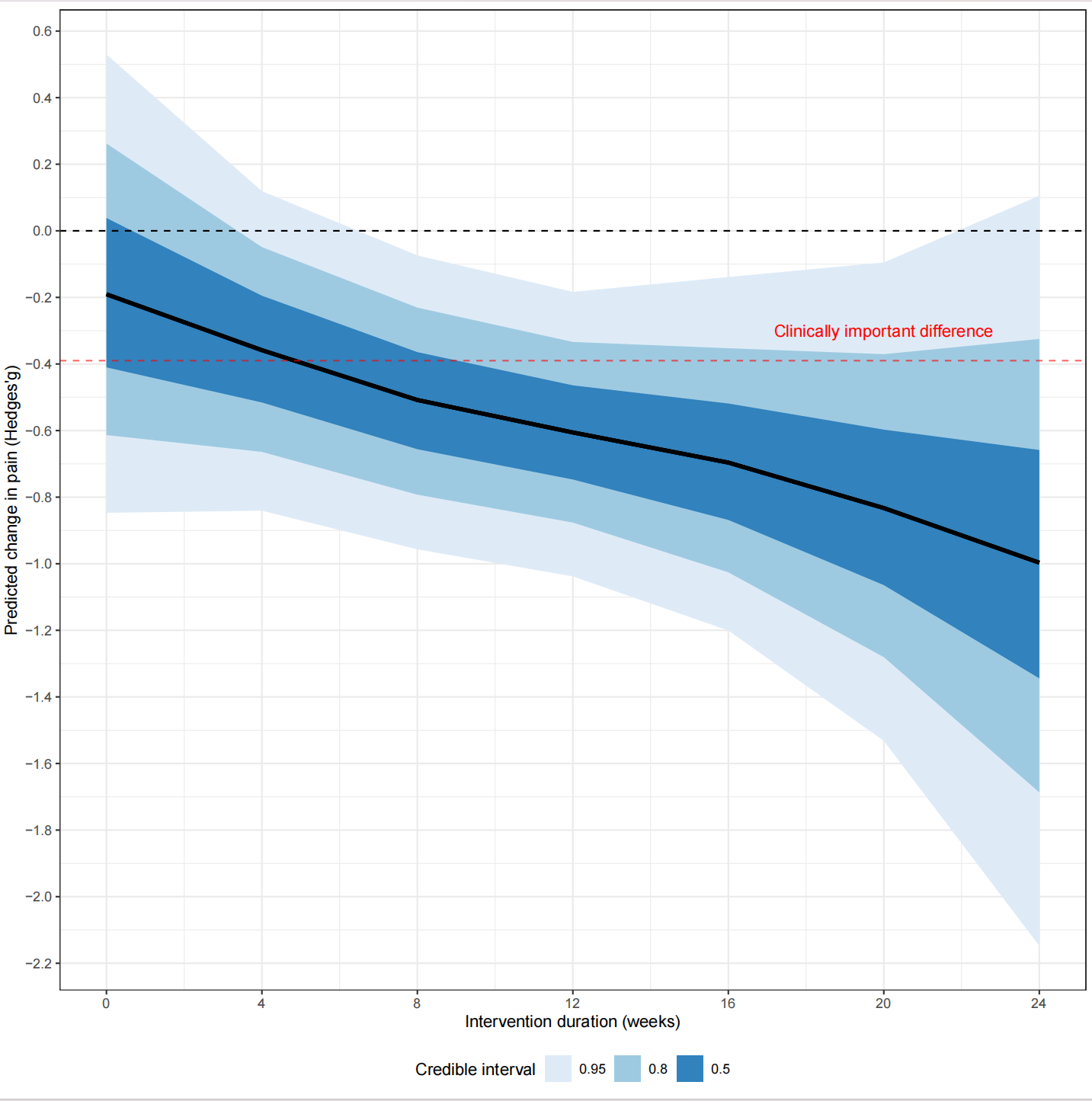


**Figure 5(c) Dose-response relationship of exercise intervention duration to migraine**


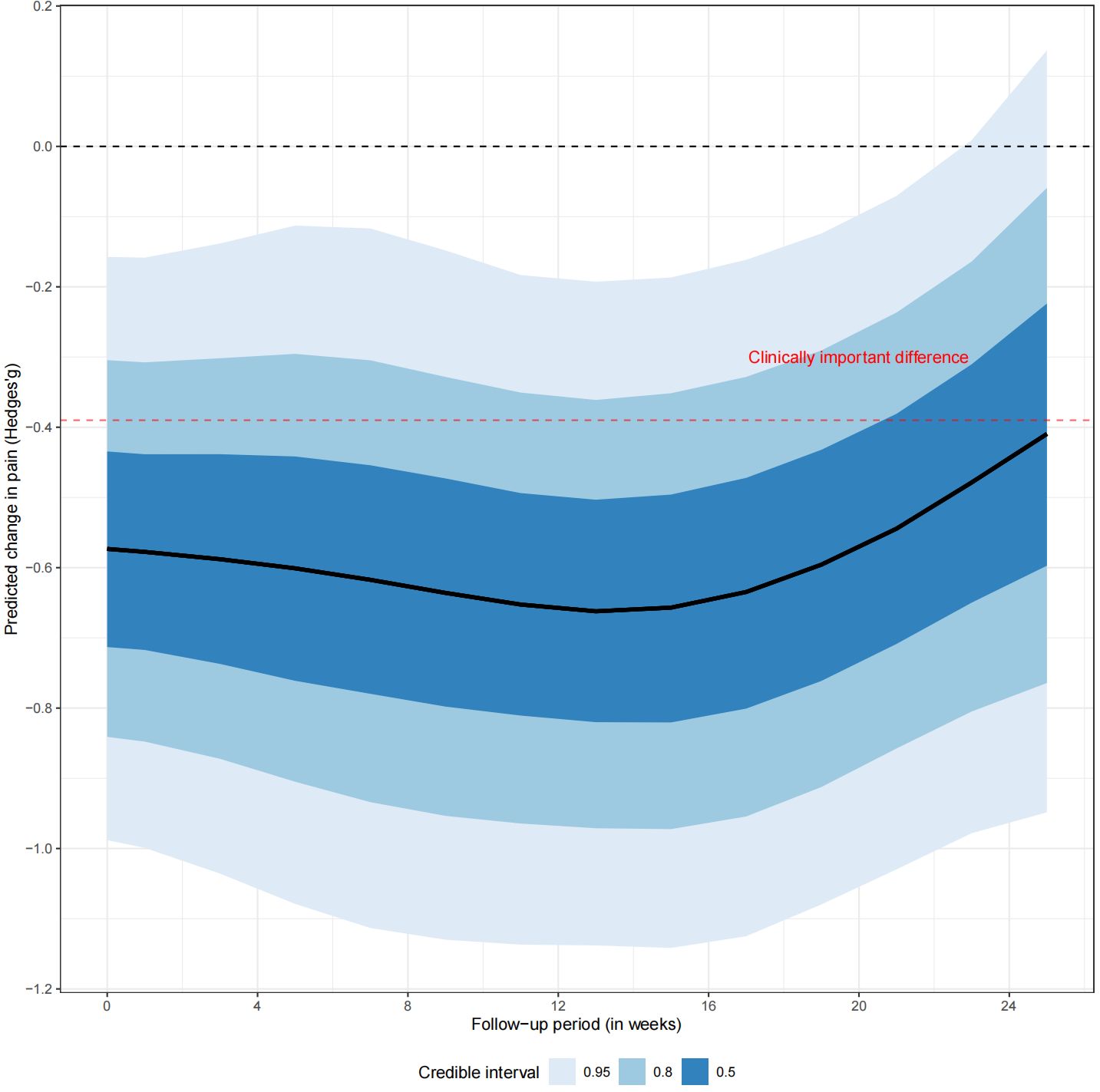


**Figure 5(d) Dose-response relationship of exercise follow-up period to migraine**

**Supplementary 10: Sample of arm-based forest plot**


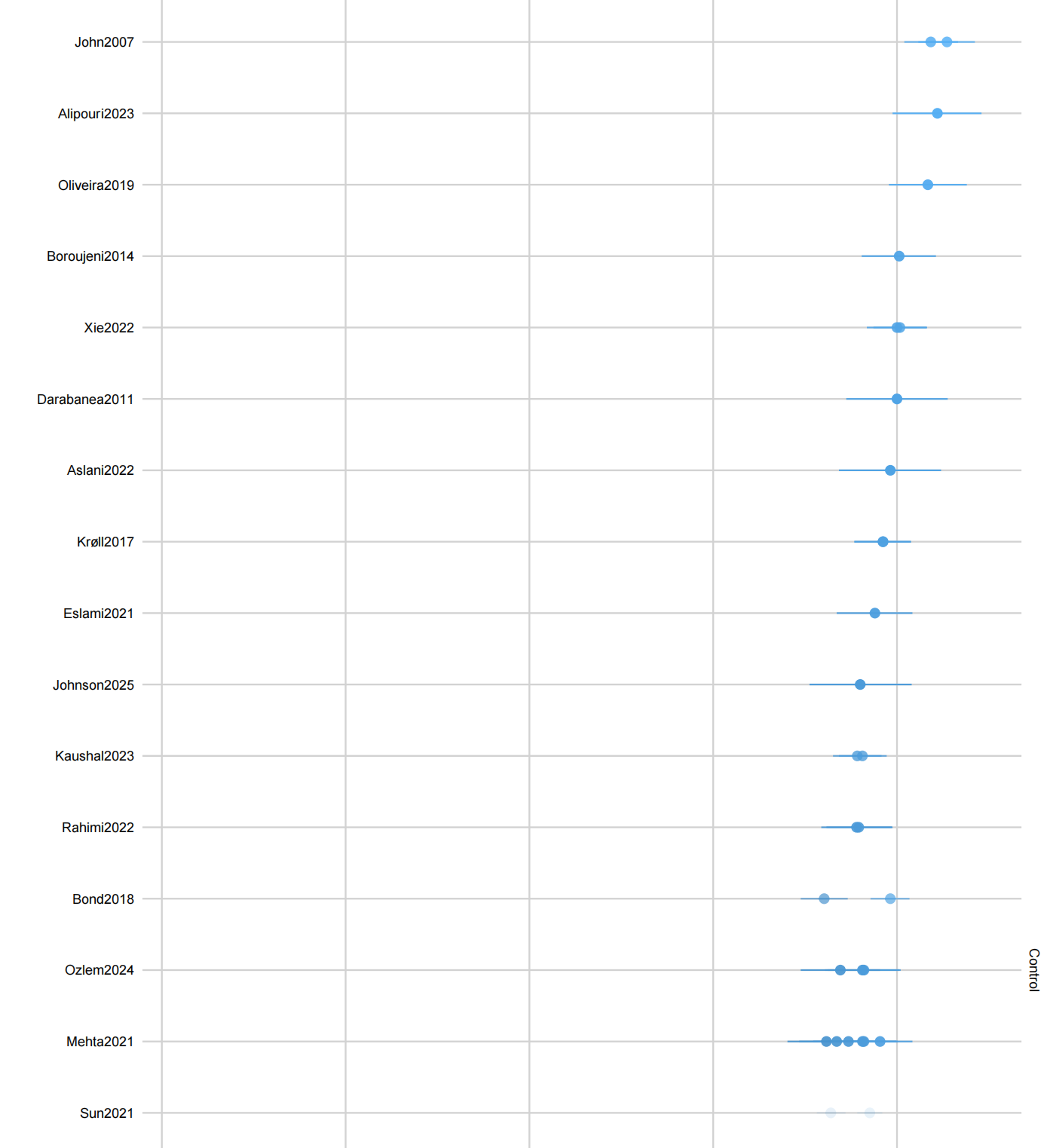


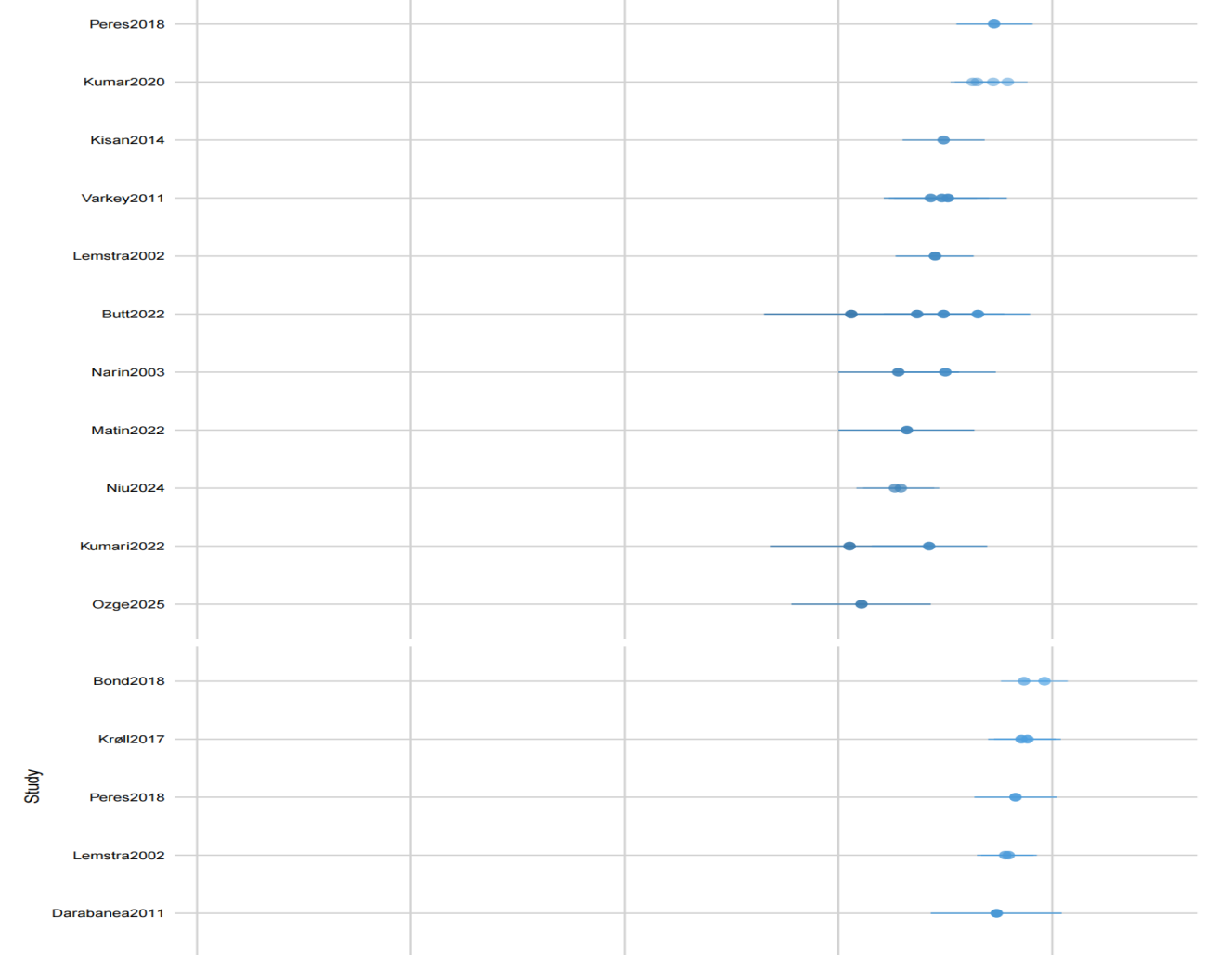


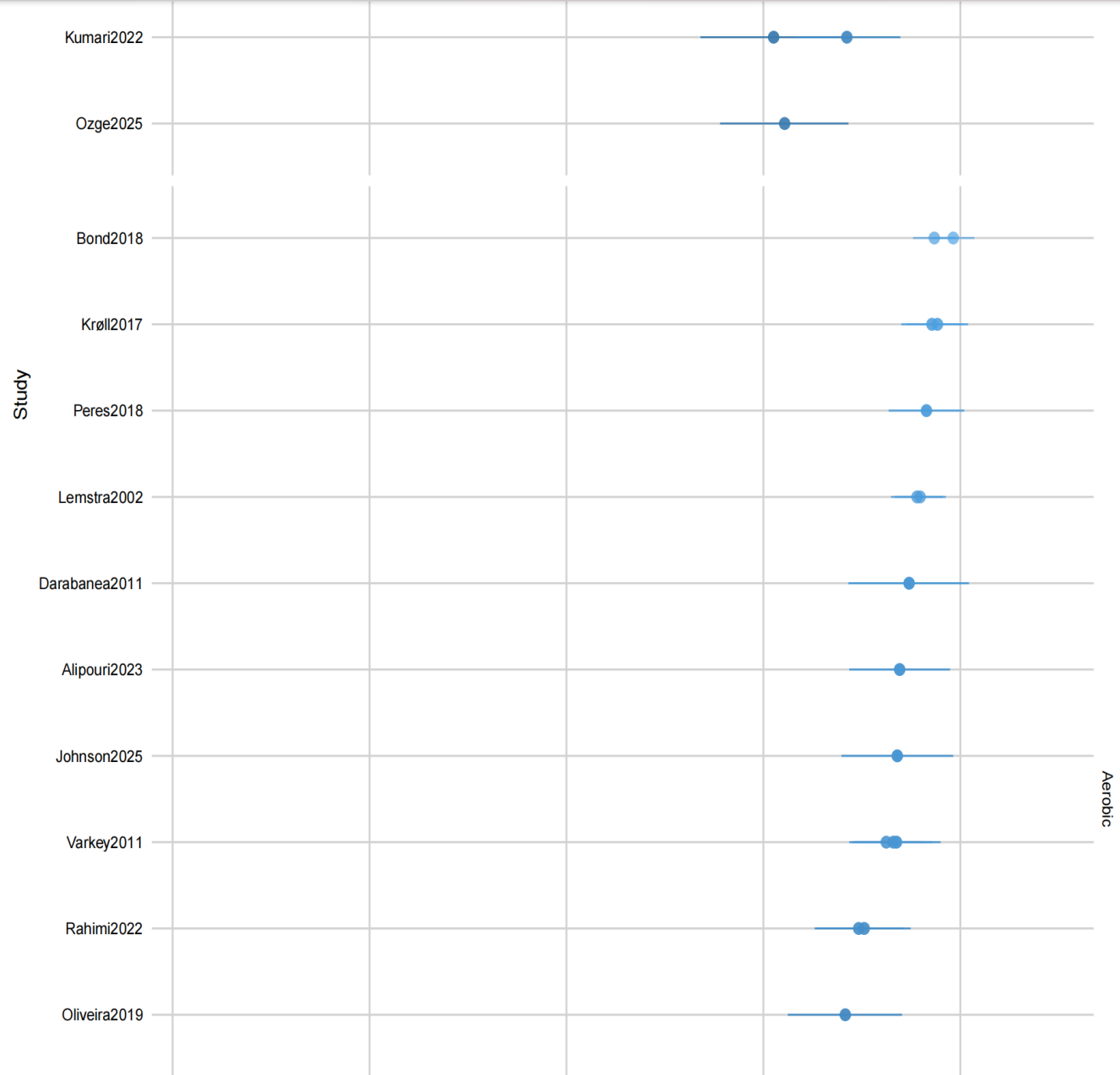


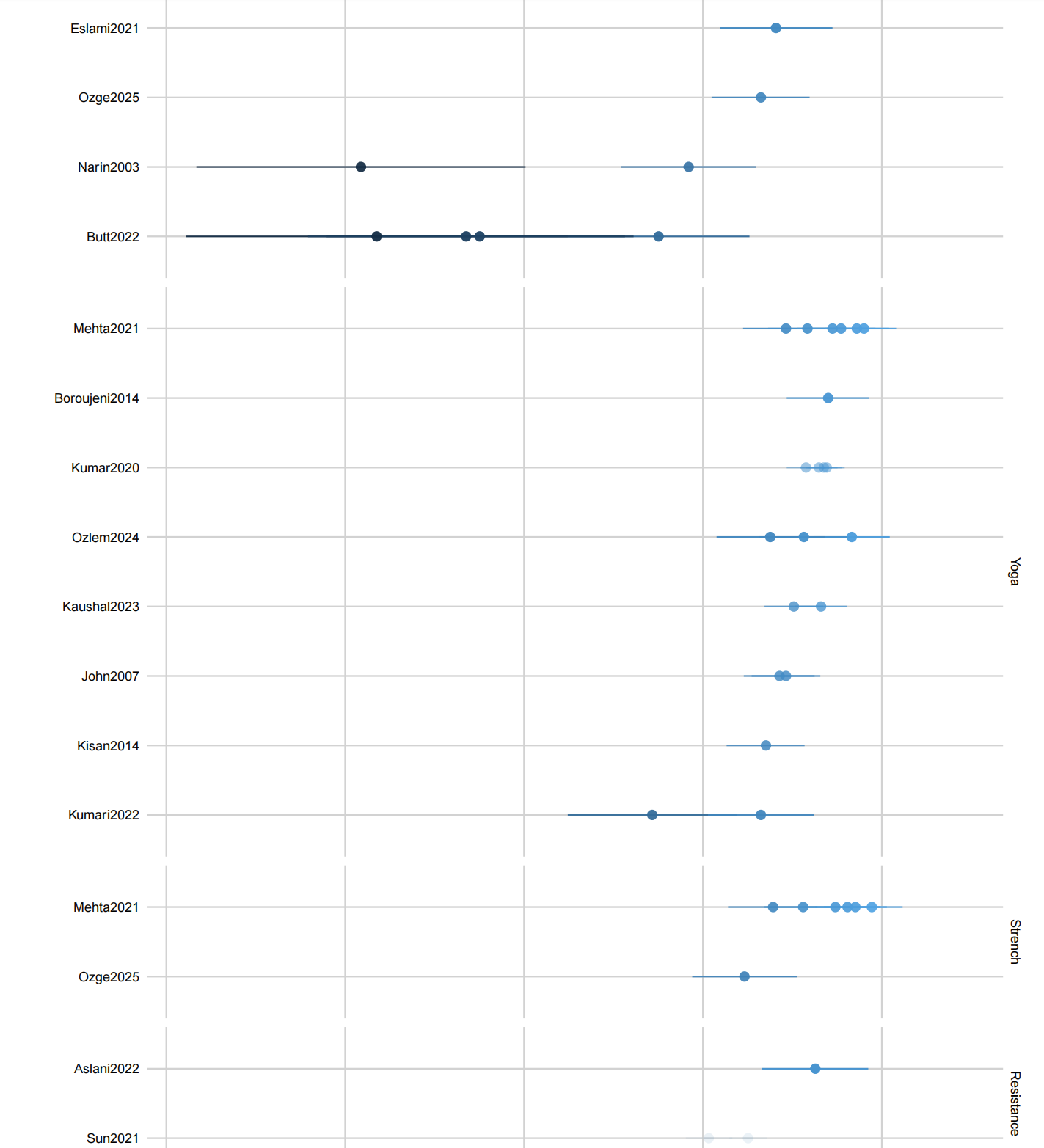


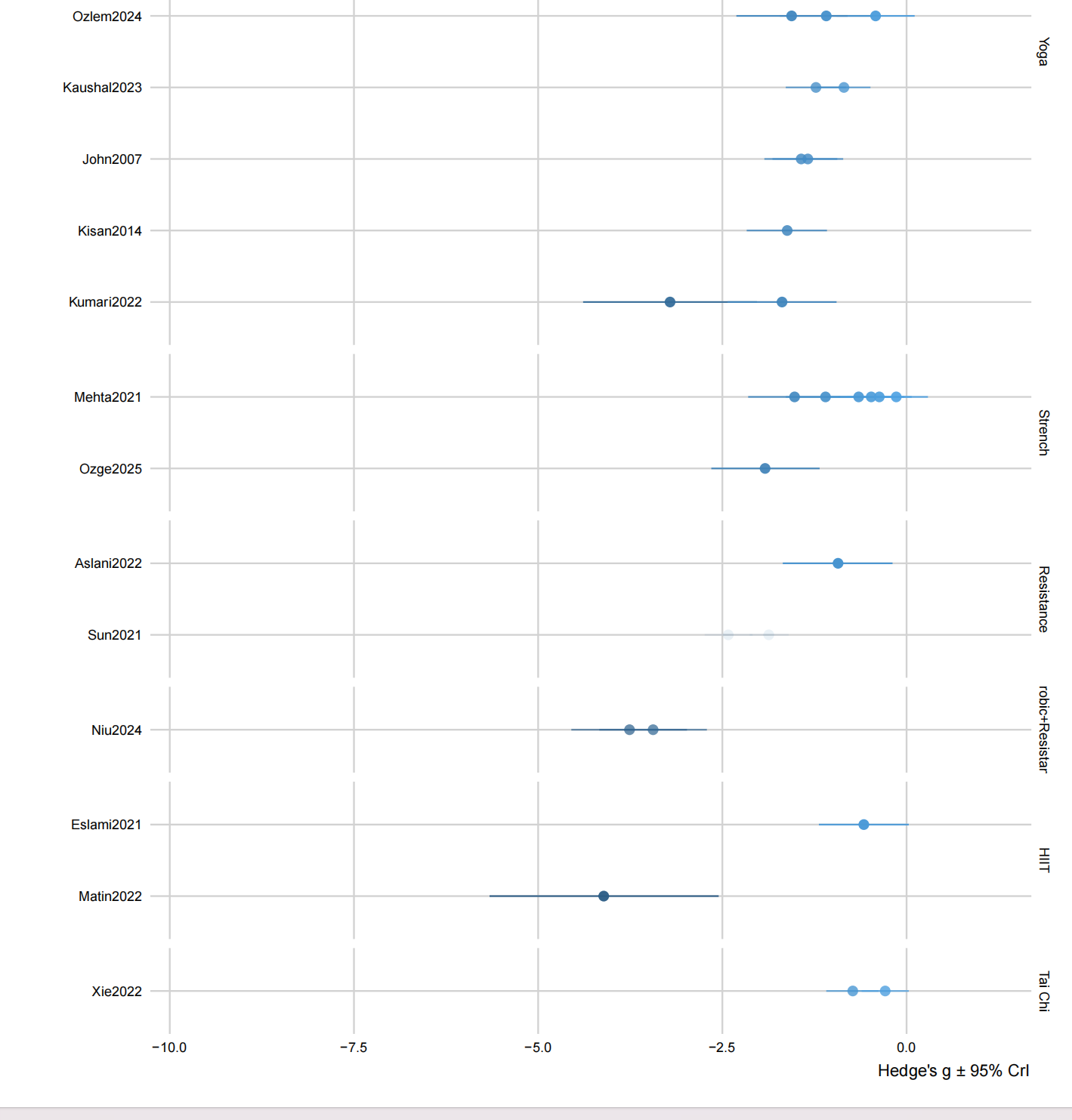


**Figure S6 Sample of arm-based forest plot**

**Supplementary 11: Model fitting effect**


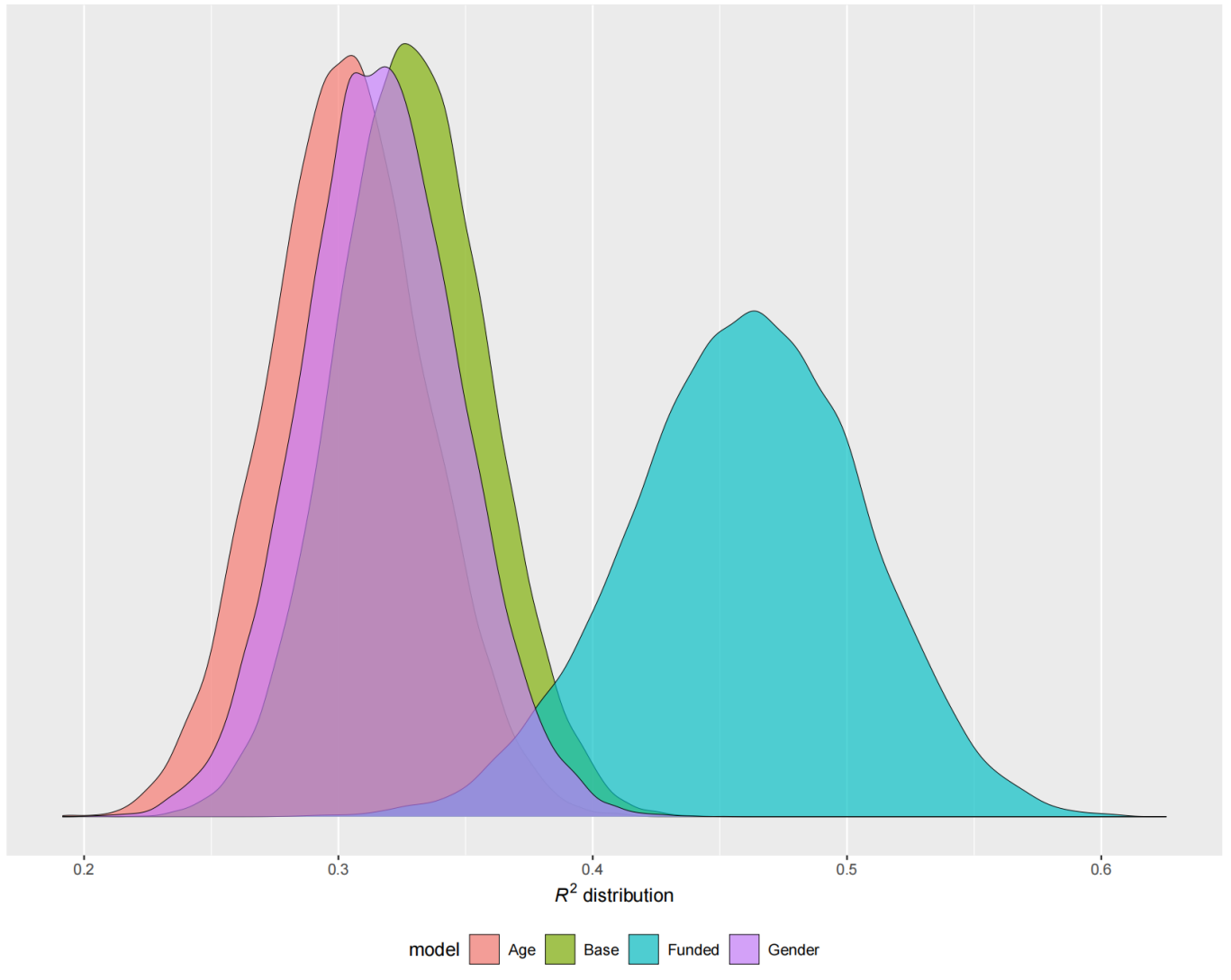


**Figure S7 Density plots for R2 from models with interactions between treatment and moderators**

**Reference**

1. Alipouri M, Amiri E, Hoseini R, Hezarkhani LA. Effects of eight weeks of aerobic exercise and vitamin D supplementation on psychiatric comorbidities in men with migraine and vitamin D insufficiency: A randomized controlled clinical trial. J Affect Disord. 2023;334:12-20.

2. Aslani P, Hassanpour M, Razi O, Knechtle B, Parnow A. Resistance training reduces pain indices and improves quality of life and body strength in women with migraine disorders. Sport Sciences for Health. 2022;18:1-11.

3. Bond DS, Thomas JG, Lipton RB, Roth J, Pavlovic JM, Rathier L, et al. Behavioral weight loss intervention for migraine: a randomized controlled trial. Obesity. 2018;26(1):81-7.

4. Boroujeni MZ, Marandi SM, Esfarjani F, Sattar M, Shaygannejad V, Javanmard SH. Yoga intervention on blood NO in female migraineurs. Adv Biomed Res. 2015;4:259.

5. Butt MN, Maryum M, Amjad I, Khan OJ, Awan L. Effects of aerobic exercise and progressive muscle relaxation on migraine. JPMA The Journal of the Pakistan Medical Association. 2022;72(6):1153-7.

6. Darabaneanu S, Overath CH, Rubin D, Lüthje S, Sye W, Niederberger U, et al. Aerobic exercise as a therapy option for migraine: a pilot study. Int J Sports Med. 2011;32(6):455-60.

7. Eslami R, Parnow A, Pairo Z, Nikolaidis P, Knechtle B. The effects of two different intensities of aerobic training protocols on pain and serum neuro-biomarkers in women migraineurs: a randomized controlled trail. Eur J Appl Physiol. 2021;121(2):609-20.

8. John PJ, Sharma N, Sharma CM, Kankane A. Effectiveness of yoga therapy in the treatment of migraine without aura: a randomized controlled trial. Headache. 2007;47(5):654-61.

9. Johnson KT, Zawadzki MJ, Widome R, Kavanaugh MS. Acceptability of a Combined Aerobic Exercise and Sleep Intervention for Sedentary Individuals with Migraine. International Journal of Behavioral Medicine. 2025:1-12.

10. Kaushal A, Padam A, Sharma M, Sharma S. Effect of Pranayama as Adjuvant to Medical Treatment on Severity, Frequency, and Duration of Headache in Migraine Patients: An Open-Label Randomized Controlled Trial. Annals of Indian Academy of Neurology. 2023;26(5).

11. Kisan R, Sujan M, Adoor M, Rao R, Nalini A, Kutty BM, et al. Effect of Yoga on migraine: A comprehensive study using clinical profile and cardiac autonomic functions. Int J Yoga. 2014;7(2):126-32.

12. Krøll LS, Hammarlund CS, Linde M, Gard G, Jensen RH. The effects of aerobic exercise for persons with migraine and co-existing tension-type headache and neck pain. A randomized, controlled, clinical trial. Cephalalgia. 2018;38(12):1805-16.

13. Kumar A, Bhatia R, Sharma G, Dhanlika D, Vishnubhatla S, Singh RK, et al. Effect of yoga as add-on therapy in migraine (CONTAIN): A randomized clinical trial. Neurology. 2020;94(21):e2203-e12.

14. Kumari S, Dhar M, Pathania M, Kumar N, Kulshrestha P, Singh A. Yoga as an Adjuvant therapy in management of migraine- An open label randomised trial. J Family Med Prim Care. 2022;11(9):5410-6.

15. Lemstra M, Stewart B, Olszynski WP. Effectiveness of multidisciplinary intervention in the treatment of migraine: a randomized clinical trial. Headache. 2002;42(9):845-54.

16. Matin H, Taghian F, Chitsaz A. Artificial intelligence analysis to explore synchronize exercise, cobalamin, and magnesium as new actors to therapeutic of migraine symptoms: a randomized, placebo-controlled trial. Neurological Sciences. 2022;43(7):4413-24.

17. Mehta JN, Parikh S, Desai SD, Solanki RC, A GP. Study of Additive Effect of Yoga and Physical Therapies to Standard Pharmacologic Treatment in Migraine. J Neurosci Rural Pract. 2021;12(1):60-6.

18. Narin SO, Pinar L, Erbas D, Oztürk V, Idiman F. The effects of exercise and exercise-related changes in blood nitric oxide level on migraine headache. Clin Rehabil. 2003;17(6):624-30.

19. Niu N, Hao Y, Cui Y, Li M. Effects of aerobic and resistance exercises on psychological and cognitive functions in patients with post-stroke migraine. Top Stroke Rehabil. 2024:1-9.

20. Oliveira AB, Ribeiro RT, Mello MT, Tufik S, Peres MFP. Anandamide Is Related to Clinical and Cardiorespiratory Benefits of Aerobic Exercise Training in Migraine Patients: A Randomized Controlled Clinical Trial. Cannabis Cannabinoid Res. 2019;4(4):275-84.

21. Baykan Çopuroğlu Ö, Çopuroğlu M. Multistrategic Approaches in the Treatment of Acute Migraine During Pregnancy: The Effectiveness of Physiotherapy, Exercise, and Relaxation Techniques. Medicina (Kaunas). 2024;61(1).

22. Dündar Ö, Can S, Eliaçık S, yıldırım t. The Effects of Hatha-Yoga Based Exercises Program on Pain with Migraine in Women: A Randomized Controlled Study2024.

23. Fernando Prieto Peres M, Prieto Peres Mercante J, Belitardo de Oliveira A. Non-Pharmacological Treatment for Primary Headaches Prevention and Lifestyle Changes in a Low-Income Community of Brazil: A Randomized Clinical Trial. Headache. 2019;59(1):86-96.

24. Rahimi MD, Hassani P, Kheirkhah MT, Fadardi JS. Effectiveness of eye movement exercise and diaphragmatic breathing with jogging in reducing migraine symptoms: A preliminary, randomized comparison trial. Brain Behav. 2023;13(1):e2820.

25. Sun L, Li G, Liu F, Wang Y, Zhang L, Minoret C. Resistance exercise relieves symptoms of vestibular migraine patients with MRI diagnosis: A randomized parallel-controlled single-blind clinical trial. Revue neurologique. 2022;178(4):370-6.

26. Varkey E, Cider A, Carlsson J, Linde M. Exercise as migraine prophylaxis: a randomized study using relaxation and topiramate as controls. Cephalalgia. 2011;31(14):1428-38.

27. Xie YJ, Tian L, Hui SS-C, Qin J, Gao Y, Zhang D, et al. Efficacy and feasibility of a 12-week Tai Chi training for the prophylaxis of episodic migraine in Hong Kong Chinese women: A randomized controlled trial. Frontiers in Public Health. 2022;Volume 10 - 2022.
